# An Unusual and Protracted Course of a Haggitt 3 Malignant Polyp Recurrence

**DOI:** 10.7759/cureus.54731

**Published:** 2024-02-22

**Authors:** Pratik Raichurkar, Tae Jun Kim, Christopher Byrne

**Affiliations:** 1 Colorectal Surgery, Royal Prince Alfred Hospital, Sydney, AUS

**Keywords:** colorectal cancer surveillance, endoscopic mucosal resection, general surgery, colorectal cancer screening, colorectal cancer recurrence

## Abstract

Timely detection of colorectal cancer recurrence is paramount, as treatment of early-stage recurrence greatly improves survival and outcomes. Current guidelines outline post-resection surveillance through endoscopy, CT imaging, and tumor markers for five years; however, there is minimal data to guide follow-up beyond this. We present the case of a 60-year-old female with locoregional recurrence 15 years after endoscopic mucosal resection of a low-grade Haggit level 3 sigmoid colon polyp. Unusually the recurrence was noted as an incidental finding following investigation of an elevated alpha-fetoprotein level post liver transplant, and a retrospective review of imaging revealed a calcified sigmoid mesentery mass. While surgical pathology revealed locoregional recurrence, there was no evidence of this on surveillance and preoperative colonoscopy. Through this case, we discuss the risk factors for late recurrence of colorectal cancer whilst exploring the literature and guidelines around this subset of patients. As new guidelines are developed, it may be important to consider late recurrence and individualize follow-up regimes based on risk factors.

## Introduction

As colorectal cancer (CRC) screening programs have become more robust and readily available, the incidence of malignant colonic polyps continues to increase. Up to 5% of endoscopically resected adenomas will demonstrate malignancy [1]; however, despite this, there is a controversy in the literature and management of these malignant polyps (MP). Decisions regarding formal oncological resection and surveillance are ultimately based on the risk of locoregional lymphatic spread. This risk is based on the level of carcinoma differentiation, lymphovascular invasion, completeness of endoscopic resection margin, and level of submucosal invasion [2,3]. Patients with low-risk features and low Kikuchi (Sm1) or Haggitt levels (1-3) (i.e. invasion confined to the polyp stalk, and not colonic submucosa) can commonly be managed with endoscopic mucosal resection alone, with minimal risk of nodal involvement [3]. Post-resection surveillance regimes assess for locoregional recurrence and metasynchronous disease through interval endoscopy at three to six months one year, three years, and five years [4,5], which will determine the need for surgical resection. The majority of CRC recurrence occurs within the first five years and current society guidelines focus on this timeframe [4,6], however, there is a lack of data regarding surveillance beyond this, and the regimes for T1 malignant colorectal cancers continue to be discussed.

We present a case of a 60-year-old woman who underwent a clear endoscopic resection of a Haggitt level three malignant polyp which has recurred in a slow-growing, protracted course over 15 years, undetectable on endoscopy.

## Case presentation

A 60-year-old woman underwent colonoscopy and endoscopic resection of a pedunculated sigmoid polyp in 2008. Pathology revealed a 40mm tubulovillous adenoma with high-grade dysplasia and carcinoma that invaded the polyp stalk consistent with Haggitt level 3, with a margin clear by 1.02mm. Staging computed tomography (CT) imaging at the time revealed no lymph node involvement or distant metastatic spread, and follow-up endoscopy at three months, one year, three years and five years revealed no recurrence.

Subsequently, she underwent a liver transplant in 2021 for end-stage liver failure secondary to alcoholic cirrhosis, requiring mycophenolate and tacrolimus for post-transplantation immunosuppression. She had recovered well post-transplantation, otherwise, the patient’s history was significant for completely excised cutaneous squamous cell carcinoma and stage 1 melanoma. The patient reported no clinical symptoms of CRC recurrence, i.e., changes in bowel habits, abdominal pain, or weight loss.

**Figure 1:**
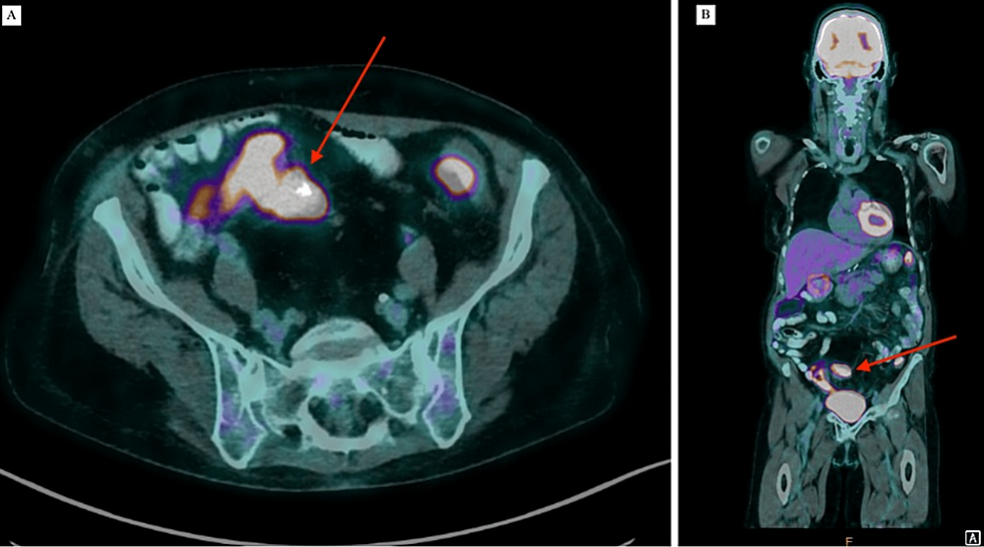
FDG-PET scan 2023, axial (A) and coronal (B) views demonstrating a 30x37mm avid mass within the sigmoid mesentery. FDG-PET: Fluorodeoxyglucose Positron Emission Tomography

**Figure 2:**
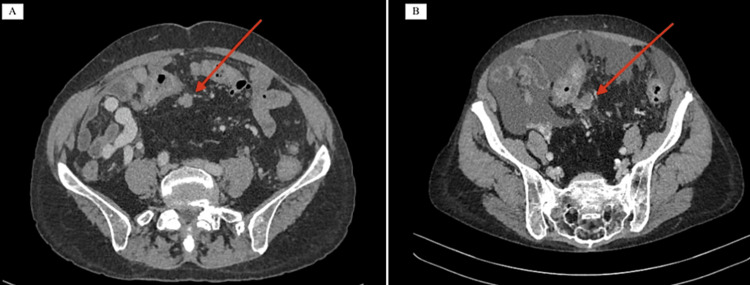
CT imaging from 2016 (A) demonstrating an unreported non enhancing mass in the sigmoid mesentery, CT imaging from 2021 (B) demonstrating progression of the mass with calcifications, and ascites CT: Computed Tomography

Two years after the transplant a newly raised routine alpha-fetoprotein level (44 µg/L), without a personal or graft history of hepatocellular carinoma triggered further investigations. A liver-specific ultrasound and CT scan were performed and reported no abnormalities, however, a subsequent multidisciplinary review revealed a potential sigmoid mesenteric mass. A positron emission tomography (PET) scan (Figures 1A, 1B) was performed, and this demonstrated a 30x37mm glucose-avid (SUV max 15) mass within the sigmoid mesentery, as well as diffuse uptake throughout the entire colon. A retrospective review of the patients’ pre-transplant CT imaging noted the presence of an unreported 20mm calcified nodule within the sigmoid mesentery present in 2016 and 2021 (Figures 2A, 2B). A staging CT demonstrated partial calcification of the mesenteric nodule and no distant lung or liver metastases. A colonoscopy was performed which did not demonstrate any mucosal lesions or recurrence, and preoperative tumor makers including carcinoembryonic antigen, CA-125, and chromogranin A was normal.

After a multidisciplinary meeting, a decision was made to proceed to colectomy in 2023. Surgery was performed laparoscopically by a senior colorectal surgeon, perioperative sigmoidoscopy revealed no mucosal abnormalities while intraoperative frozen section biopsy of the sigmoid lesion revealed adenocarcinoma, and a subsequent high anterior resection was performed. Histopathology from the sigmoid colon revealed a moderately differentiated adenocarcinoma with perineural and venous invasion, present within the submucosa, muscularis propria and subserosa. The overlying colonic mucosa was normal. None of the 14 lymph nodes showed evidence of malignancy giving a stage of T3N0M0. Given a clear PET scan and a favorable pathology, no adjuvant therapy was prescribed. The patient subsequently had an unremarkable, uncomplicated recovery, and is planned for surveillance CT imaging in one year, as well as annual serum CEA, CA19-9, and CA125 testing.

## Discussion

Currently surveillance protocols, including those from the American Society of Clinical Oncology (ASCO) [6], The National Institute for Healthcare and Excellence (NICE), and The Association of Coloproctology of Great Britain and Ireland (ACPGBI) [2] describe a follow-up period for malignant polyps for three to five years. These protocols vary but consist of six monthly physical examinations and CEA, staging CT scans, and surveillance colonoscopy. While the five-year risk of recurrence for T1 colon cancer is now below 7%, and the mean time to recurrence is under three years [7], there is a push for less intensive surveillance for these patients [8]. Following this five-year period, if no recurrence is noted, surveillance protocols follow that of the general population. 

However, this case represents an extremely protracted course of locoregional recurrence, some 15 years after excision, undetectable by colonoscopy or CEA screening. While the overall rate of delayed (>10 years) recurrence for colon cancer is quite low, there are currently no guidelines for the surveillance of malignancy polyps beyond five years. There have only been a few retrospective analyses examining recurrence beyond five years, Cho et al. analyzed 1,136 patients with resected CRC, of which only 1.2% demonstrated recurrence after five years, with only one of these patients demonstrating T1 disease [9]. Other studies have reported incidences of less than 1% for >5-year recurrence [10], the majority of these are also distant metastases within the lung or liver [11]. The main risk factors for delayed recurrence include left-sided tumors, low preoperative CEA, >4mm invasion, and male sex [9-11]. While some of these factors were present in this case, another factor may have been immunosuppression. Immunosuppression after solid organ transplantation is known to be associated with a high incidence of CRC compared to the general population, with the largest trials demonstrating a standardized incidence ratio of 1.12 [12]. Although there is a general concern that transplantation may trigger a recurrence of an occult malignancy, there is minimal data regarding liver transplantation in patients with a history of CRC. The majority of data is sourced from case reports or small retrospective trials. A review of five single-center studies with a total of 16 patients with pre-liver transplant CRC revealed an 18.8% incidence of recurrence [13], which remains higher than the general population. Given the paucity of data and comparatively low incidence of recurrence, CRC surveillance in liver transplant patients follows that of pre-transplant guidelines [14,15].

The decision to proceed with endoscopic resection compared to surgery for T1 MP is dependent on the risk of lymph node metastases and the ability to perform an en-bloc resection. Haggitt et al. described a classification system for submucosal invasion, with level 1 corresponding to invasion of the head of a pedunculated polyp; level 2 involving the polyp neck; level 3 corresponding to malignant cells within the stalk, and level 4 indicating invasion to the depth of the bowel submucosa [16]. Endoscopically resected lesions with Haggitt levels 1-3 have minimal risk of lymph node metastases, however, some studies have demonstrated a 6.2%-8% risk of lymph node involvement with Haggitt 3 lesions [17]. Generally, low-risk lesions with Haggitt levels 1-3, low-grade dysplasia, no lymphovascular invasion, and clear resection margins (>1mm) can be managed endoscopically [17]. However, there is little data on longer-term (>5 years) follow-up and rates of locoregional recurrence following EMR. The rate of locoregional recurrence following EMR is approximately 15%-18%, however, en-bloc resections of pedunculated lesions have been shown to have recurrence rates under 1% [18]. Almost 98% of local recurrence occurs within 12 months and this is where current literature and guidelines are focused. A 76-patient series published by Cui et al. demonstrated a locoregional recurrence rate of 10.7% between five and 10 years [19], while a smaller 25-patient series by Freeman demonstrated no locoregional recurrence in 10 years of follow-up post-EMR, however, 8% of patients developed metasynchronous disease [20]. Patients undergoing EMR may represent a special subgroup that may require surveillance for longer than the current guidelines recommend. However, larger studies would need to be performed to assess specific risk factors in this group.

## Conclusions

Locoregional CRC recurrence beyond five years is an uncommon occurrence, this case represents a rare, difficult-to-detect, protracted course of recurrent stage T1N0M0 CRC, discovered incidentally as part of liver transplant surveillance. Whilst the overall incidence of delayed recurrence is low, there is minimal data to guide cancer surveillance beyond five years. We propose that future guidelines consider the risk factors for long-term recurrence, and clinicians individualize follow-up duration, intervals, and protocols accordingly.
